# Magnetic Beads inside Droplets for Agitation and Splitting Manipulation by Utilizing a Magnetically Actuated Platform

**DOI:** 10.3390/mi14071349

**Published:** 2023-06-30

**Authors:** Jr-Lung Lin, Pei-Pei Hsu, Ju-Nan Kuo

**Affiliations:** 1 Department of Mechanical and Automation Engineering, I-Shou University, Kaohsiung 84001, Taiwan; ljl@isu.edu.tw (J.-L.L.); pphsu@isu.edu.tw (P.-P.H.); 2 Department of Automation Engineering, National Formosa University, No. 64, Wenhua Rd., Yunlin 63201, Taiwan

**Keywords:** magnetic manipulation, digital microfluidics, agitation, splitting, washing

## Abstract

We successfully developed a platform for the magnetic manipulation of droplets containing magnetic beads and examined the washing behaviors of the droplets, including droplet transportation, magnetic bead agitation inside droplets, and separation from parent droplets. Magnetic field gradients were produced with two layers of 6 × 1 planar coils fabricated by using printed circuit board technology. We performed theoretical analyses to understand the characteristics of the coils and successfully predicted the magnetic field and thermal temperature of a single coil. We then investigated experimentally the agitation and splitting kinetics of the magnetic beads inside droplets and experimentally observed the washing performance in different neck-shaped gaps. The performance of the washing process was evaluated by measuring both the particle loss ratio and the optical density. The findings of this work will be used to design a magnetic-actuated droplet platform, which will separate magnetic beads from their parent droplets and enhance washing performance. We hope that this study will provide digital microfluidics for application in point-of-care testing. The developed microchip will be of great benefit for genetic analysis and infectious disease detection in the future.

## 1. Introduction

Nucleic acid-based diagnostics is rapidly expanding and has various applications in infectious disease diagnosis, pharmacogenomics, oncology, and genetic testing. Miniaturized total analysis systems (μTAS) for genetic assays have been developed by using microfluidic technologies, which integrate nucleic acid extraction, amplification, and detection [1]. Microfluidics, i.e., continuous fluid flow or droplet-based actuation, has been introduced to develop μTAS for biomedical applications successfully. Although these two approaches are promising, their dependence on equipment for point-of-care testing (POCT) remains a major challenge in real-world biosample tests. In such applications, droplet-based chips have shown significant potential for storing and processing samples and reagents without the aid of pumping devices. Recently, a considerable amount of attention has been paid to droplet-based microfluidics, also known as digital microfluidics. Droplets not only serve as reaction chambers and transportation units but also as self-contained systems. In addition to their simplicity, portability, and ability to store samples and reagents on chips, droplet-actuated platforms are particularly useful for POCT. Droplet movement is effectively controlled by numerous actuated methods, including electrowetting on dielectric [2], thermocapillary force [3], surface acoustic waves [4], electrophoresis [5], optical force [6], and magnetic force [7,8,9,10,11,12]. Among all actuation devices, magnetic-actuated droplet manipulators [7,8,9,10,11,12] exhibit some advantages due to their flexibility, long distances, high driving forces, and easy use. Using droplets as micro-reactors, reagents can be transported, merged, mixed, and analyzed effectively [13,14].

In contrast to agarose, sepharose, or silica beads, magnetic beads are manipulated by using magnetic forces to facilitate and accelerate their capture and washing. In general, magnetic beads consist of a magnetic core and a non-magnetic coating for selectively bonding biomaterials, such as cells, proteins, or DNA fragments [15]. Magnetic separation can be used for the concentration and purification of biosamples. In addition to being applied as an actuated medium, magnetic beads are used to carry biomolecules; superparamagnetic particles, for example, can be utilized to bind and transport nucleic acids [16]. Magnetic beads have been successfully applied to DNA separation [17], protein digestion [18], immunoassays [19], and cell capture [20] and are therefore attractive from a practical perspective. Traditionally, the general operation of magnetic beads is described as follows: (1) Magnetic beads suitable for specific antibodies are chosen. (2) The magnetic beads are added into biosamples or buffers. (3) The desired virus binds specifically to the magnetic beads conjugated with antibodies. (4) The analyte-bound beads are captured by using an external magnet. (5) The analyte is eluted from beads after crude sample components are washed away. Such a traditional method is laborious and requires high amounts of reacting samples and reagents to detect diseases effectively. Later, to facilitate the automatic transport and mixing of magnetic beads, microfluidic devices, i.e., micropumps and micromixers, were integrated into continuous flow schemes for the transport and biosample capturing of magnetic beads. Planar coils embedded on-chip are also employed to generate magnetic fields that attract magnetic beads. In particular, PCR thermal cycles are applied to embed planar coils on chips [21]. The continuous flow scheme for effective purification and enrichment, however, wastes a massive amount of biosamples, reagents, and buffers.

Instead of using the continuous scheme for bioapplications, magnetic beads surrounded by droplets were prepared to solve the problems mentioned above. Currently, several actuations, i.e., permanent magnets [8,9] or planar coils [7,10,11,12], were used to transport, split, and merge droplets containing magnetic beads. Consequently, this approach significantly shortened time and reduced costs when trial-and-error methods were used for genetic analysis. A variety of commercially available reagents and surface-coated particles have been utilized to perform on-chip DNA extraction and purification from crude biosamples [7,8,9,11,12]. Microparticle encapsulation, sequential fusion, rapid mixing, and dilution have successfully demonstrated in conventional droplet-based microfluidics. In continuous microfluidics, unbound reagents are washed from magnetic particles by serial dilution. Recently, advanced on-chip techniques have been successfully demonstrated for multistep binding and washing steps on magnetic particles by traversing multilaminar reagent streams [22,23,24]. Recently, millimeter-sized ‘ferrobots’ were tested for precise manipulation of magnetized droplets and delivery of workflows related to nucleic acid amplification [25]. However, despite recent advancements in magnetic handling, isolating magnetic beads for bioassays can be difficult due to the limited reconfigurability and compartmentalization of magnetic beads. Moreover, the short traversing times between multilaminar flow streams limit increasing incubation time with reagents for postprocessing. Therefore, a robust and simple washing step on a digital droplet-based microfluidics platform for magnetic particle-based assays is in high demand. In contrast to continuous flow and electrowetting-based microfluidic systems, in-droplet microparticle separation systems have rarely been investigated.

In this study, we developed a magnetically actuated droplet platform that utilizes magnetic beads actuated by a coil array-induced magnetic field gradient for agitation under the aid of neck-shaped barriers for separating operations. The use of these two mechanisms facilitates the washing of magnetic beads. The combination of two-layer 6 × 1 array coils and topographical barriers enables the simplified, effective, and fully automated manipulations of droplets for sample purification.

## 2. Theoretical Analysis

Microparticle kinematics obeys Newton’s second law, which depends on the balance among z-axial magnetic force (*F_B__,z_),* Stokes’ force (*F_St_*), gravity force (*F_g_*), and buoyancy force (*F_b_*). It can be expressed as follows:

(1) $$m_{b}\frac{du_{b}(t)}{dt}=F_{B,z}-F_{g}-F_{St}+F_{b}$$

Droplet movement is driven by the *x*-axis magnetic force (*F_B,__z_*), which can be expressed by [26]

(2) $$F_{B,z}=\frac{m_{b}\;\chi \;B_{m}B_{Z,Max}}{\mu_{o}\rho_{b}D_{d}}$$

Here, *m_b_* and *ρ_b_* are the mass and density of the clustered magnetic beads, respectively; *D_d_* is the diameter of the droplet, and *B_m_* is the *z*-axial magnetic field generated by the Helmholtz coil. Moreover, the maximum *z*-direction magnetic field (*B_z,Max_*) is generated by the coil applied with a given DC current.

The movement of the microparticle in the surrounding medium is governed by Stokes’ law. Stokes’ force (*F_St_*) is expressed as follows:

(3) $$F_{St}=3\pi \eta d_{b}u_{b}(t)$$

where *η* is the medium viscosity, *d_b_* is the microparticle diameter, and *u_b_* is the microparticle velocity.

The difference between the gravity force and buoyancy force of a microparticle is expressed as follows:

(4) $$\Delta F_{g,b}=\Delta \rho gV_{b}$$

Here, *Δρ* and *V_b_* represent the difference between the densities of a microparticle and medium and the volume of a microparticle, respectively.

Substituting Equations (2)–(4) into Equation (1) yields

(5) $$m_{b}\frac{du_{b}(t)}{dt}=\frac{\chi m_{b}B_{m}\nabla B_{z}}{\rho_{b}\mu_{o}}-\frac{\Delta \rho gm_{b}}{\rho_{b}}-3\pi \eta d_{b}u_{b}(t)$$

Equation (5) can be simplified as

(6) $$\frac{du_{b}(t)}{dt}-\alpha u_{b}(t)=\Phi_{m}$$

Equation (6) can be integrated as

(7) $$u_{b}(t)=\frac{\Phi_{m}}{\alpha }\left(1-e^{-t/S_{t}}\right)$$

Here, $\alpha =\frac{1}{S_{t}}={\left(\frac{168\pi^{2}\eta^{3}}{\rho_{b}m_{b}^{2}}\right)}^{1/3}$, $\Phi_{m}=\frac{\chi B_{m}\nabla B_{z}}{\rho_{b}\mu_{o}}\mp \frac{\Delta \rho }{\rho_{b}}g$. The parameters used in this study are as follows: *η* = 10^−3^ pa−s; *m_b_* = 300 µg; *μo* = 4π × 10^−7^ Tm^−1^; χ = 0.235; *B_m_* = 50.0 mT; *B_z,Max_* = 4.885 mT; *z* = 3.0 mm *ρ_b_* = 1500 kg m^−3^; Δ*ρ* = 500 kg m^−3^. Thus, the coefficients of α and *Φ_m_* can be obtained as α = 22.79 s^−1^; *S_t_* = 0.044 s; *Φ_m_* = 6.08 ms^−2^.

(8) $$u_{b}(t)=\frac{\Phi_{m}}{\alpha }\left(1-e^{-t/S_{t}}\right)$$

The velocity of the microparticles inside droplets is expressed as follows:

(9) $$u_{b}(t)=\left\{\begin{array}{cc} \frac{\Phi_{m}}{\alpha }\left(1-e^{-t/S_{t}}\right) & 0\leq t\leq \frac{\tau }{2}\\ \frac{\Phi_{m}}{\alpha }\left(1-e^{-(T-t)/S_{t}}\right) & \frac{\tau }{2}\leq t\leq \tau \end{array}\right.$$

Here, τ is the periodic time.

## 3. Design and Experiments

### 3.1. Design and Fabrication

**Figure 1:**
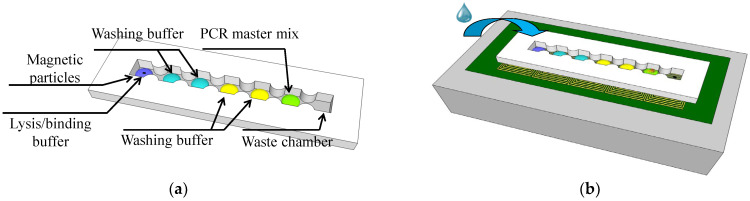
(**a**) Cartridge layout with seven compartments on a planar coil array and (**b**) composition of the liquid in each compartment along with the droplet movement sequence.

The on-chip method for cell lysis and nucleic acid extraction and purification adhered to a standard protocol and used silica-coated magnetic particles. A small amount of surfactant (Span-80, Sigma-Aldrich, Burlington, MA, USA) was introduced into the mineral oil (M5904, Sigma-Aldrich, Burlington, MA, USA) coating on the cartridge to prevent droplet splitting. Sessile reagent droplets were sequentially added to load each compartment. The first compartment of the device received a droplet of biosample mixed with lysis buffer, Tris-EDTA, proteinase K, and magnetic particles. Subsequently, compartments 2 through 6 were loaded in sequence with four individual washing buffers and the PCR reagent mixture [11]. The last compartment was purposefully left empty to serve as a waste collection reservoir. The cartridge is illustrated in Figure 1a. In this study, the magnetically actuated chip consisted of three layers, namely, a cartridge, coil chip, and cooling system, as shown in Figure 1b. The cartridge was designed with seven compartments. A neck-shaped channel interconnected every compartment. During each washing step, the microparticles merged with the wash buffer then advanced to the next reagent droplet. A magnetic field gradient induced by a planar coil was employed to control the magnetic particles on this platform. A cooling system was used to dissipate the high DC current-induced thermal heat.

**Figure 2:**
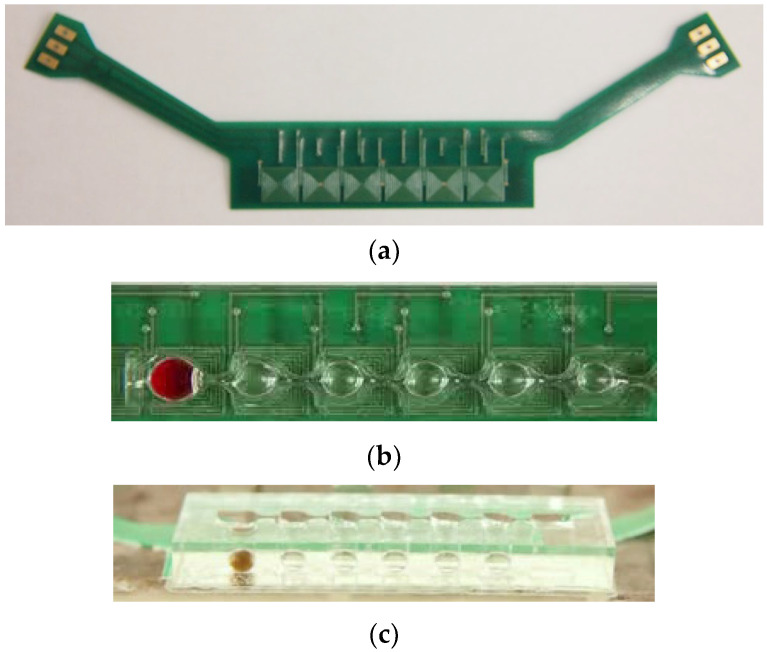
(**a**) Coil array chip and layout of droplet cartridge integrated with two-layer coils. (**b**) Top-side view and (**c**) lateral side view of the cartridge.

We used the printed circuit board (PCB) technique to print a two-layer 6 × 1 coil array chip to generate the magnetic field gradients for particular applications. The coil chip contained six coils on its top and bottom layers with a distance of 200 μm, as shown in Figure 2a. The square-profile coils were designed with eight windings, and the coils were designed with a width and spacing of 150 μm and partially overlapped between adjacent coils. Master molds were created by using 3D rapid prototyping and acryliconitrile butadiene styrene, then used to cast poly-dimethylsiloxane (PDMS) prepolymer (Sylgard 184, Dow Corning Corporation, Midland, MI, USA) with a base-to-crosslinker ratio of 9:1. After the device had been cured at 80 °C for 30 min, it was attached to a glass coverslip by using a standard O_2_ plasma treatment process. We dip-coated the device with a solution of 1% *w*/*w* Teflon AF 1600 (DuPont Corp., Wilmington, DE, USA) in FC-40 solvent (3M Company, St. Paul City, MN, USA) and baked it overnight at 80 °C to prevent biomolecule surface adsorption and to prevent reagent droplets from spreading on the cartridge surface. The cartridge, which was composed of seven compartments interconnected serially by six sieve structures, were bound onto the two-layer 6 × 1 coil array chip. Figure 2b,c shows the top-side and lateral side views of the cartridge, respectively.

### 3.2. Experiments

**Figure 3:**
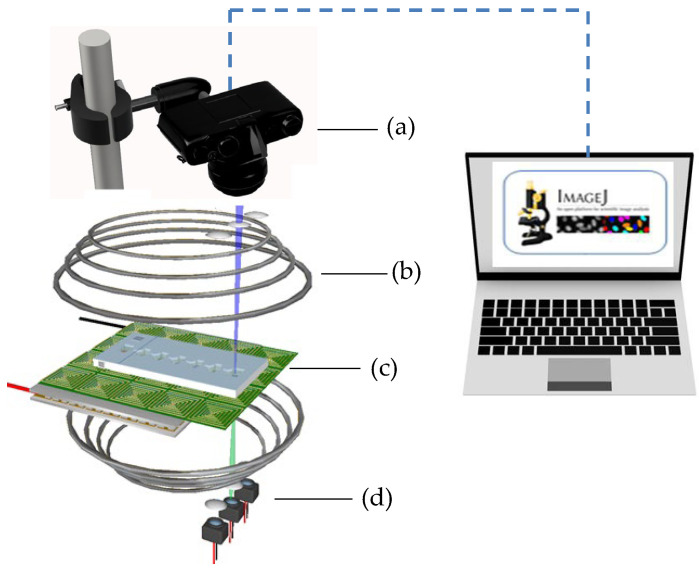
Experimental setup of the magnetically actuated platform. a: digital camera incorporating a CCD; b: Helmholtz coil; c: magnetically actuated chip; d: cooling system.

The experimental setup (Detailed in Supplementary Document S1), as depicted in Figure 3, included a Helmholtz coil laid out around the magnetically actuated chip to generate a uniform transverse magnetic field of approximately 50.0 mT. The magnetically actuated chip was placed on an experimental platform designed to observe microparticle kinematics. A diagnosed droplet containing 2.88 μm magnetic particles (MF-DEX-3000, MagQu LLC, Surprise City, AZ, USA) was added to the first compartment of the cartridge. Six transparent liquid droplets were sequentially added into the specific compartment of the cartridge. The magnetic field’s magnitude and direction can be controlled by adjusting the DC currents, whereas the magnetic fields produced by each coil were managed by a custom-designed analog circuit. A digital camera connected to a charge-coupled device (CCD, Cool SNAP HQ2, Photometrics, Huntington Beach, CA, USA) was used to capture images of droplet movement and magnetic particle kinetics for further analysis. In addition, the images of the dye were converted into grayscale values by using ImageJ (v1.8.0, National Institute of Health, Bethesda, MD, USA). Custom software developed in LabVIEW (National Instruments, Austin, TX, USA) was applied to oversee the magnitude, duration, and sequence of the DC currents. A crucial component of this setup is the thermoelectric cooler integrated with a K-type thermocouple that functioned as a cooling system beneath the PCB. We used a PID controller with a thermoelectric module to maintain a consistent 37 °C, crucial for cell-friendly conditions. This system effectively counteracts Joule heating, ensuring a steady temperature during the experiment [12].

## 4. Results and Discussion

### 4.1. Magnetic Characterization

The theoretical calculation of the z-axial magnetic field of the eight wound coils is difficult and complex. Thus, a simple model is introduced that uses a single coil for calculation. For different heights, *B_Z__,Max_* at the center of the coil can be expressed as follows: (Detailed in Supplementary Document S2).

(10) $$B_{Z,Max}=\frac{4\mu_{o}I}{4\pi }\sum_{i=1}^{N=8}\frac{2l_{i}^{2}}{\left(l_{i}^{2}+z^{2}\right){\left(2l_{i}^{2}+z^{2}\right)}^{1/2}}$$

Here, *z* represents the height at different locations, either A or B. li signifies the distance of the i-th winding coil from the center. The distance can be calculated using the formula *l_i_* = 225 + (*i* − 1) × 300 μm. In the present study, *z_A_* = 300 μm at location A, and *z_B_* = 400 μm at location B. After substituting these values into Equation (10), we can obtain *B_Z,A_* = 3.66 × I for location A and *B_Z,B_* = 3.10 × I for location B, respectively.

**Figure 4:**
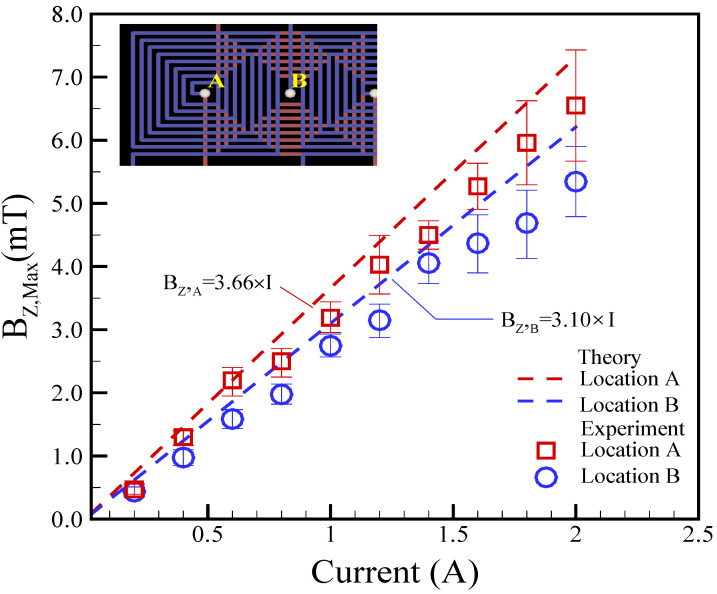
Experimental magnetic field (*Bz,_Max_*) versus different applied currents at different locations. Here, z_A_ = 300 μm at location A and z_B_ = 400 μm at location B.

The magnetic field in the z-direction (*B_Z,Max_*) was theoretically predicted by Equation (10), and the obtained results were then compared with the measurements. The dynamics of the magnetic field generated by the coil is a crucial issue for the magnetically actuated mechanism of droplets. Consequently, understanding the magnetic field is a priority for understanding the underlying physical phenomenon. A tesla meter (tm-401, Kanetec, Tokyo, Japan) was employed to measure the magnetic fields at the center of the coil. Figure 4 presents the maximum experimental magnetic field (*Bz,_Max_*) in relation to various applied currents at different locations. Locations A and B represent the top and bottom layers of the coil, respectively, as indicated in Figure 4. The height difference between the two locations of A and B was set to be approximately 100 μm. In experiments involving planar coils of varying heights (i.e., different locations), the magnetic fields exhibited a quasi-linear correlation with the applied DC currents. As anticipated, the applied DC current and magnetic field demonstrated a linear relationship. The magnetic fields also decreased with the increase in coil height, emphasizing the importance of considering the coil’s position when designing and optimizing the magnetically actuated mechanism for droplet control. Furthermore, the theoretical analysis was reasonably in agreement with the experimental measurements. The theoretical predictions were within 17.2% and 25.7% of the experimental measurements for locations A and B, respectively.

The 1-D, i.e., *z*-directional, steady-state heat conduction equation can be expressed as

(11) $$\frac{\partial }{\partial z}\left(k\frac{\partial T}{\partial z}\right)+\dot{g}=0$$

The power density ($\dot{g})$ of the coil is obtained as

(12) $$\dot{g}=\frac{\dot{Q}}{V}=\frac{I^{2}R}{tA_{S}}=\frac{L_{C}\times I^{2}}{t\sigma A_{S}A_{C}}$$

Here, σ and *A_C_* are the electrical conductivity and cross-sectional area of a winding copper, respectively. *A_S_* and *t* are the surface area and thickness of a coil, respectively. The top and bottom surfaces dissipated by heat convection are expressed as

(13) $$-\frac{k\nabla T}{2}=h(T_{S}-T_{\infty })$$

where *k* and *h* are the thermal conductivity and heat convention coefficient, respectively.

Substituting Equation (12) into Equation (11) and integrating yields the following formula: (Detailed in Supplementary Document S3).

(14) $$T_{S}=\frac{L_{C}}{4\sigma hA_{S}A_{C}}I^{2}+T_{\infty }$$

In this study, the geometric parameters were calculated as *L_C_* = 17.4 mm, *A_S_* = 36 mm^2^, *Ac* = 5.25 × 10^−3^ mm^2^, *σ* = 5.996 × 10^7^ Sm^−1^ There are several factors that affect the convection coefficient *h*, such as flow pattern, fluid properties, surface geometry, bulk velocity, and temperature difference between surfaces and their surroundings [27]. In general, the convection coefficient (*h*) ranges from 2 to 25 Wm^−2^ K^−1^ [28]. So, it is difficult to be determined. Here, *h* = 10 and 15 Wm^−2^K^−1^ are conducted to investigate the surface temperature.

**Figure 5:**
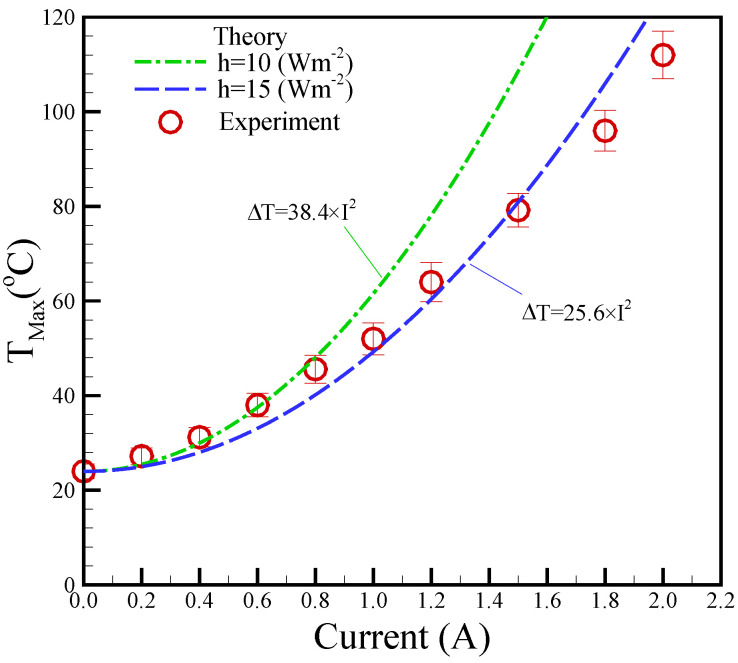
Experimental maximum temperature (*T_Max_*) versus different applied currents. The surrounding temperature (*T_∞_*) is 25 °C.

A higher magnetic field was achieved under higher applied DC currents. However, higher applied DC currents can also generate increased thermal power, leading to elevated temperatures. Therefore, temperature management is a critical aspect of the biosample platform, making temperature control an essential challenge. Figure 5 displays the maximum temperature measured in relation to the applied current. K-type thermocouples were affixed to the tops of the planar coils to measure their temperatures. In the experimental results, the maximum temperatures exhibited a quadratic increase with the increase in applied currents, emphasizing the importance of maintaining a balance between magnetic field strength and temperature control. The theoretical predictions based on the experimental data agreed with the experimental data with a variation of 12.8% for the convention h = 15 Wm^−^^2^K^−^^1^.

### 4.2. Agitation Characterization

**Figure 6:**
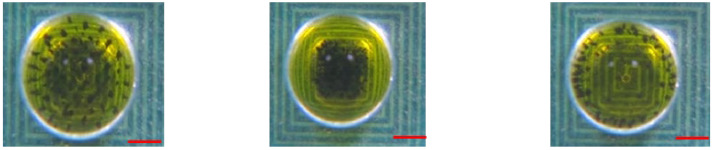

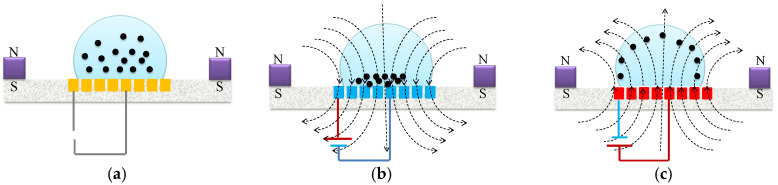
Illustrations depicting the (**a**) release, (**b**) attraction, and (**c**) repulsion of magnetic beads within a droplet. The red scalar represents 1.0 mm.

In the present study, we set a standard reference scenario by keeping a steady direct current of 1.5 A, alternating at a frequency of 1.0 Hz, and enclosing a 100 μg bead in a 20-uL droplet at a consistent temperature of 37 °C. Our goal was to examine and understand the fundamental behavior of the washing process under these particular conditions. By agitating magnetic particles under alternating attractive and repulsive forces, the coil platform can actuate magnetic particles efficiently (Figure 6). Permanent magnets were used to affix the magnetic polarization of the microparticles in a particular direction. Repulsion and attraction were created as a result of changing the direction of the DC current. The droplet was agitated by a single coil that was centered over it. The coil generated magnetic field gradients that were alternatively upward or downward due to the change in the direction of the DC current. The microparticles inside the droplet can be repeatedly agitated by alternating the direction of the current. During this study, 300 μg of microparticles were agitated with an applied DC current of 1.5 A at a frequency of 1.0 Hz.

**Figure 7:**
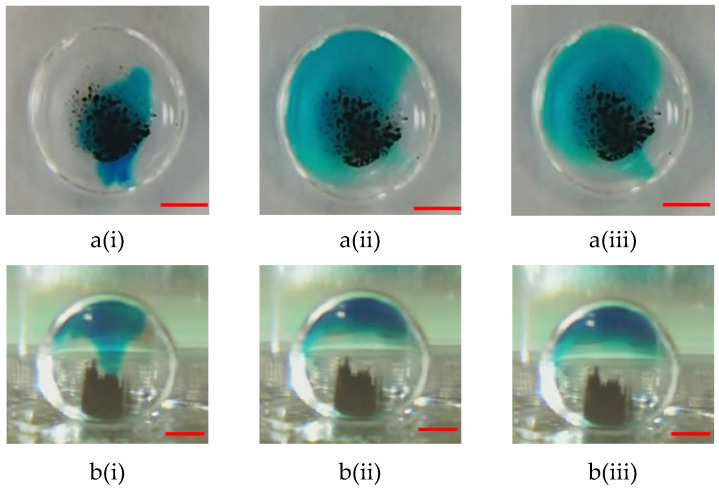
(**a**) Top-side and (**b**) lateral side views of photographic sequences showing mixing performance under agitation in the absence of magnetic beads at the different times of (**i**) t = 0 s; (**ii**) t = 25 s, and (**iii**) t = 50 s. In this case, the 2.88-μm magnetic beads have a mass of 100.0 μg, and no DC current is applied to the single coil. The red scalar represents 1.0 mm.

**Figure 8:**
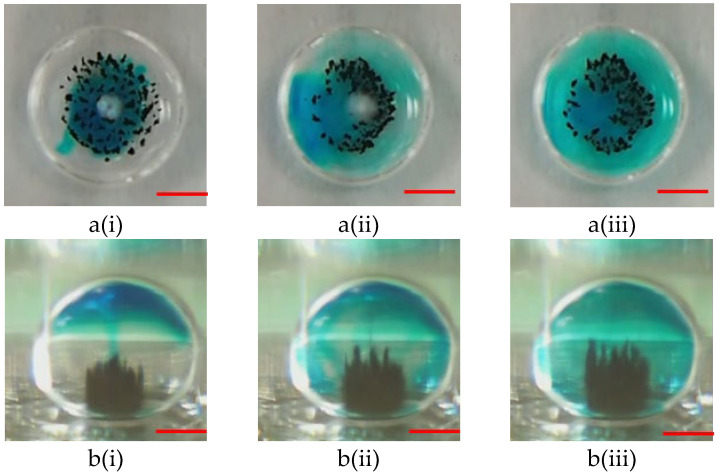
(**a**) Top-side view and (**b**) lateral sides views of photographic sequences showing mixing performance under magnetic bead agitation at the different times of (**i**) t = 0 s; (**ii**) t = 2.0 s, and (**iii**) t = 4.0 s. The conditions for this operation are as follows: a DC current of 1.5 A, a 2.88-μm magnetic beads’ mass of 100.0 μg, and an alternate frequency of 1.0 Hz. The red scalar represents 1.0 mm.

The motion of microparticles within droplets can cause a significant mixing effect. Thus, microparticles can effectively perform capturing and washing functions. Two samples containing blue dye (oil phase) and a buffer solution (sodium borate, 1 mM, pH 8.2) were used to evaluate the mixing effect to verify the mixing efficiency of the proposed magnetically actuated mixer. First, 5.0 μL of blue dye was pipetted into a 15 µL droplet of buffer solution, as shown in Figure 7a(i) and Figure 8a(i). As can be clearly seen in the figures, no microbeads were agitated under the initial conditions. In the droplet-based mixer, the blue dye (oil phase) mainly diffused in the upper part of the droplet (see Figure 7a(ii),(iii) but cannot diffuse in the lower part because the density of oil was less than that of water (see Figure 7b(ii),(iii)). Conversely, significant mixing was observed in Figure 8, when a DC current of 1.5 A with a frequency of 1.0 Hz was applied to the coil. Figure 8 shows the images taken successively by agitating magnetic beads to disturb the fluid inside droplets. By moving the magnetic beads back and forth, the fluid inside the droplets was disturbed to distribute the dye uniformly after 4.0 s. Figure 8b(ii),(iii) illustrate that the homogeneous mixing of the blue dye resulted in high mixing efficiency. A droplet-based mixer was agitated in four back-and-forth cycles to demonstrate uniform mixing quickly by using magnetic beads. The experimental results were consistent with previously reported findings [12].

### 4.3. Splitting Characterization

**Figure 9:**
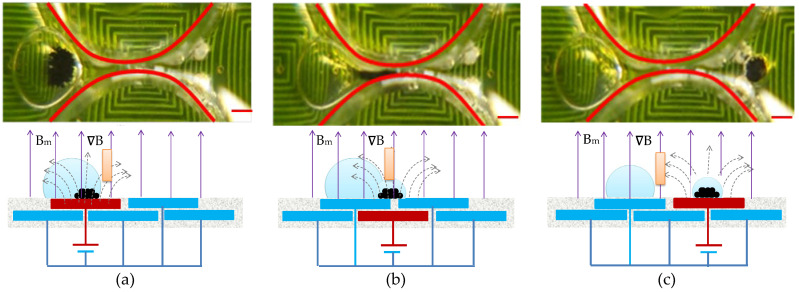
Photographic sequences showing droplet splitting assisted by a sieve structure. (**a**) Magnetic beads congregated at the droplet’s rim; (**b**) Aggregated magnetic beads traversing through the topological shape; (**c**) Clustered magnetic beads dispersing to create a satellite droplet. The established operational parameters are as follows: a DC current setting of 1.5 A, beads with a size of 2.8 μm and a collective mass of 100.0 μg within the parent droplet, and an alternating frequency of 1.0 Hz for the nearby coil. The red scalar represents 1.0 mm.

**Figure 10:**
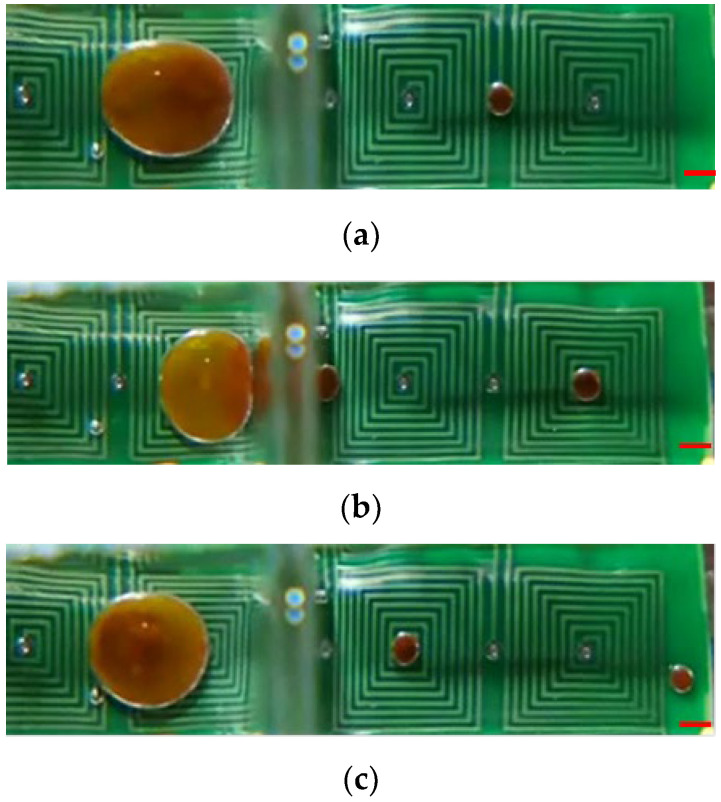
Satellite droplets continuously split from a parent droplet through a microfluidic orifice. (**a**) Splitting of a single satellite droplet; (**b**) Splitting of two satellite droplets; (**c**) Transportation and collection of two satellite droplets. The established operational parameters are as follows: a DC current setting of 1.0 A, beads with a size of 1.0 μm and a collective mass of 1000.0 μg within the parent droplet, and an alternating frequency of 1.0 Hz for the nearby coil. The red scalar represents 1.0 mm.

For the purpose of diagnosis, magnetic beads bound to a specific virus have to be split from the parent droplet. Therefore, the kinetics of magnetic bead splitting from droplets is highly challenging. Figure 9 shows how a droplet containing magnetic particles was deformed by neck-shaped channels and split into a smaller plug that contained water from its parent droplet. This was achieved by using surface topography to help the droplet split. When the maximum of the magnetic field was reached at the destination compartment, magnetic particles were gathered into a plug and pulled through an elongated neck-shaped structure until scission occurred [11]. As a result of splitting from its parent droplet, a magnetic particle joined the following droplet. With an optimal concentration of surfactant, in this case 0.5% *w*/*w* mixed with mineral oil, magnetic particles can be successfully extracted from a parent droplet and transported to the subsequent compartment, all while preventing premature merging with the preceding droplet [29]. By doing so, droplet compartments could be packed more compactly onto smaller cartridges. Magnetic particles could be collected in the center of a compartment by creating a field maximum at the center. Two distinct phenomena can occur when utilizing varying magnetic forces, i.e., applied DC current, to separate magnetic beads from the droplet. In Figure 9, the DC current of 1.5 A is employed to separate all magnetic beads from the droplet. In contrast, Figure 10 uses the DC current of 1.0 A to segregate only a portion of the magnetic beads, thereby enabling the continuous separation of satellite droplets. The continuous images show that the satellite droplets successfully separated from their parent droplets. Supplementary Video S4 presents the separation sequence of the droplets.

### 4.4. Washing Characterization

**Figure 11:**
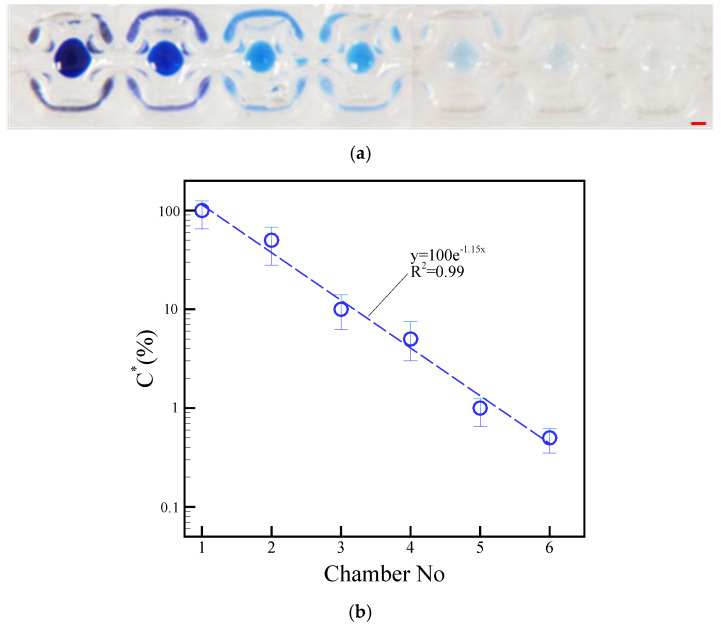
(**a**) Dye intensity in each chamber during the separation of magnetic beads from droplets. (**b**) Concentration of droplets normalized in accordance with different chambers. The operational conditions are set as follows: a DC current of 1.5 A, a beads’ mass of 100.0 μg in the droplet, and a frequency of 1.0 Hz alternation for the adjacent coil. The red scalar represents 1.0 mm.

We estimated the color intensity of the blue dye carried over between each a droplet to evaluate the effectiveness of the washing process. The neck-shaped channel had a gap of 500 μm and a length of 1500 μm. The washing process involved the motion of a droplet, agitation of magnetic beads within a droplet, and extraction of magnetic beads from a droplet. The blue dye intensity of a droplet indicates how well the magnetic beads have been washed after each step. Incomplete washing is indicated by higher intensity. By contrast, lower intensity indicates more complete washing. The effect of washing was evaluated by measuring the mean gray value of the blue dye in compartment 1 against serial dilutions of the buffer. A DC current of 1.5 A was applied to split microparticles from a droplet, and 1.5 A was applied to agitate magnetic beads inside a droplet. The driving frequency was also set to 1.0 Hz. The magnetic particles were washed in order from compartment 2 to compartment 6 after separating them from compartment 1 containing blue dye. In the first chamber, a 15 µL droplet of blue dye was dispensed. Six 15 µL transparent droplets were dispensed in the second to sixth compartments as previously shown in Figure 1a. We use a 20 μL droplet, consisting of 15 μL water and 5 μL dye, due to its compatibility with our planar coil and the visibility it offers for real-time performance analysis. A DC current of 1.5 A with an alternation frequency of 1.0 Hz was applied during eight agitation cycles to distribute the dye in each compartment uniformly. By using a DC current of 1.5 A and a frequency of 1.0 Hz, the magnetic beads inside a droplet were actuated across the neck-shaped channel to split from the previous droplet. The intensity of a blue-colored droplet depends on the magnetic beads that carry liquid dye to the next droplet. The blue dye was also uniformly distributed within the next transparent droplet through the agitation of the magnetic beads. The dye intensity of the droplet should have reduced compared with that of the previous droplet. The dye intensity images of the first droplet were compared with those of the following droplets to quantify the amount of liquid carried by the magnetic beads during the washing process. The intensity of blue dye in different chambers is shown in Figure 11a. Moreover, a graph showing the normalized concentration of blue dye in each chamber can be seen in Figure 11b. These two parameters are related by the Eq. C* = 100e^−1.15N^. The coefficient of determination was R^2^ = 0.99. Here C* represents the normalized concentration, and N represents the washing chamber. The continuous images show that the magnetic beads inside a droplet successfully separated from their previous parent droplet to perform the washing effect. Supplementary Video S5 presents the washing sequence of the magnetic beads inside the droplets.

**Figure 12:**
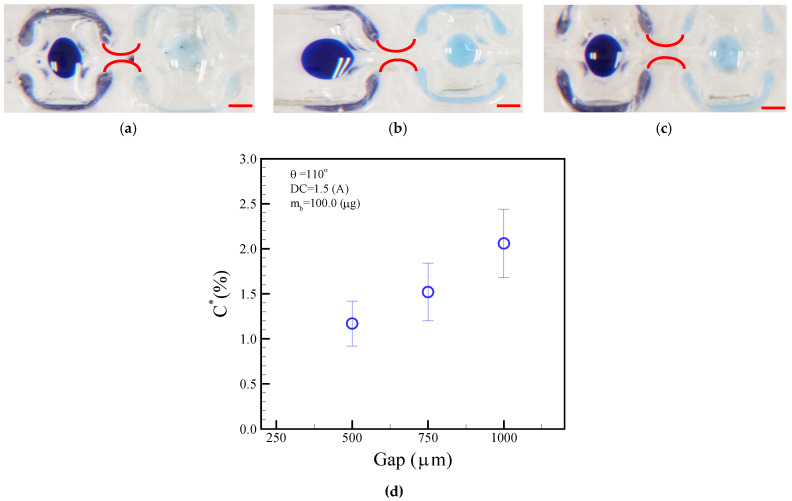
Normalized concentration of blue dye after the fourth washing process in different neck-shaped channel gaps of (**a**) 500, (**b**) 750, and (**c**) 1000 μm. (**d**) Concentration of droplet normalized in accordance with different neck-shaped gaps. The operational conditions are set as follows: a DC current of 1.5 A, a bead mass of 100.0 μg in the droplet, and a frequency of 1.0 Hz alternation for the adjacent coil. The red scalar represents 1.0 mm.

A larger neck-shaped gap allowed a droplet to pass through without magnetic bead separation. Conversely, a smaller neck-shape gap prevented the parent droplet from moving and allowed the magnetic beads to be extracted. As discussed in this section, we investigated the effect of the neck-shaped gaps on the magnetic beads’ ability to carry over droplets. Figure 12a–c show the droplet dilution diagrams obtained after the magnetic beads were split from the neck-shaped gaps of 500, 750, and 1000 μm. Analysis was performed by separating the magnetic beads from the fourth washing solution and diluting them. ImageJ software was used to determine whether any changes should be made to the color intensity of the photos, and the results were compared with a previously established standard curve. The experiments revealed variations of 1.17%, 1.52%, and 2.06% in blue dye intensities in the neck-shaped gaps of 500, 750, and 1000 μm, respectively, as shown in Figure 12d. Washing performance can be improved by a smaller neck-shape gap of 500 μm. Our analysis will provide valuable insight into the influence of neck-shaped gaps on droplet agitation and splitting in microfluidics.

**Figure 13:**
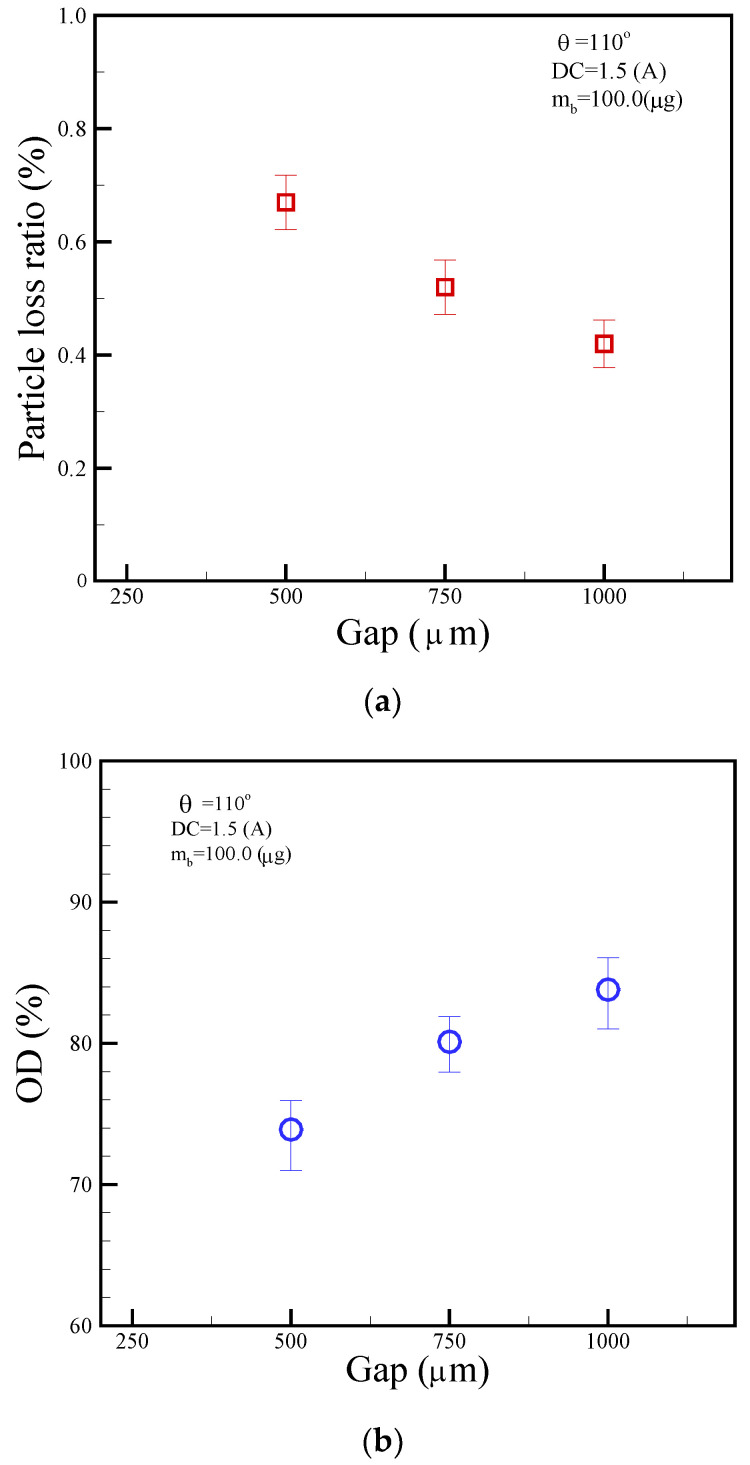
The relationship of (**a**) particle loss ratio and (**b**) optical density (OD) with respect to different gap sizes of 500, 750, and 1000 μm. The operational conditions are set as follows: a DC current of 1.5 A, a beads’ mass of 100.0 μg in the droplet, and a frequency of 1.0 Hz alternation for the adjacent coil.

Particle correction and the optical detection were set up in chamber No. 6. Particle loss plays a pivotal role when separating magnetic beads from the parent droplet, especially in microfluidic or lab-on-a-chip setups. We define the particle loss ratio as the ratio of collected particle mass to the initial particle mass. Figure 13a showed a particle-loss ratio of 0.67%, 0.52%, and 0.42% for gap sizes of 500, 750, and 1000 μm, respectively, suggesting larger gaps might enhance bead transport efficiency, reducing bead loss. Moreover, as indicated by the Beer–Lambert law, optical density (OD) or absorbance correlates directly with the concentration of the absorbent substance within a solution. Figure 13b portrays this relationship, displaying OD values of 73.8%, 80.1%, and 83.9% for gaps of 500, 750, and 1000 μm, respectively. It suggests a lower concentration of blue dye results in lower OD, whereas a higher concentration increases OD.

## 5. Conclusions

We demonstrated a magnetic droplet-based actuation system for agitating microbeads inside droplets and splitting magnetic particles from droplets. We also formulated a straightforward theoretical model for both the magnetic field and thermal temperature. This model aligns reasonably well with experimental measurements, displaying variations of 25.7% for the magnetic field and 12.8% for the thermal temperature. In addition, the use of microbeads within droplets enhanced washing performance based on agitation and separation. In the current study, particle-loss ratios of 0.67%, 0.52%, and 0.42% correspond to gap sizes of 500, 750, and 1000 μm, respectively. Concurrently, OD values are 73.8%, 80.1%, and 83.9% for the same gaps. These results demonstrate that particle loss increases with larger gaps, whereas OD increases with smaller gaps. Additionally, the proposed platform can be integrated with thermal control and optical detection, adding to its benefits. We hope to deliver the developed platform for POCT in the future.

## Data Availability

The data used in this study can be found within the article. For any further data that bolster the conclusions of this study, please direct requests to the author in charge of correspondence.

## Supplementary Materials

The following supporting information can be downloaded at: https://www.mdpi.com/article/10.3390/mi14071349/s1. Document S1: Configuration for the experimental setup of the entity; Document S2: Magnetic-field derivation of a single coil with eight turns; Document S3: Temperature derivation of a single coil with eight turns; Video S4: Satellite droplets continuously splitting from a parent droplet; Video S5: The washing performance evaluated within different chambers of a droplet.
