# Supplementary material for: Magnetic Beads inside Droplets for Agitation and Splitting Manipulation by Utilizing a Magnetically Actuated Platform
Source: Micromachines (Basel). 2023 Jun 30;14(7):1349. doi: 10.3390/mi14071349 (PMC10384566; doi:10.3390/mi14071349)
Supplement: Supplementary file 1 [file micromachines-14-01349-s001.zip › supplementary document S1.pdf]

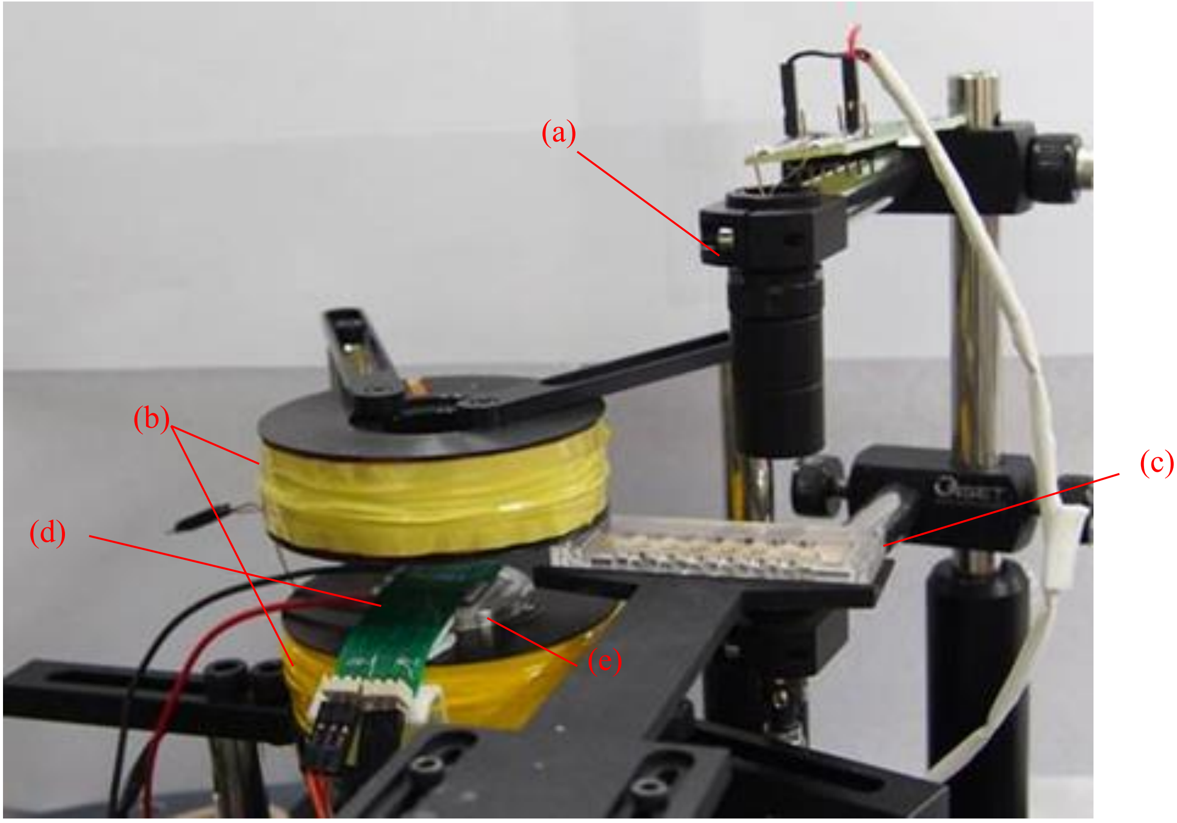

Figure S1. Experimental setup of the magnetically actuated platform. a: digital camera attached with a CCD; b: Helmholtz coil; c: Cartridge; d: a two-layer 6×1 coil array chip; and (e) a thermoelectric module.
