# Supplementary material for: Magnetic Beads inside Droplets for Agitation and Splitting Manipulation by Utilizing a Magnetically Actuated Platform
Source: Micromachines (Basel). 2023 Jun 30;14(7):1349. doi: 10.3390/mi14071349 (PMC10384566; doi:10.3390/mi14071349)
Supplement: Supplementary file 1 [file micromachines-14-01349-s001.zip › supplementary document S2.pdf]

We simplify the 8-winding as 8-loop square coils where at the same center as illustrated as Figure A.1.

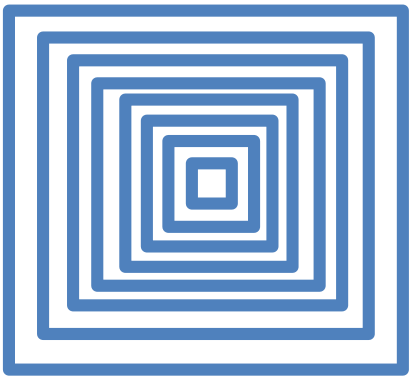

Figure A-1: A schematic representation of the eight-loop square coils.

According to Biot-Savart's law, the magnetic field dB at any point P due to element *dl* of a current-carrying wire is given by:

$$\vec{B} = \frac{\mu_o}{4\pi}\oint_C \frac{Id\vec{l}\times\vec{r}}{|\vec{r}|^{3/2}} \qquad \text{(A-1)}$$

In this case, *dl* is the vector of the current element in the direction of current flow into the square-shaped conductor (Figure A-2(a)), and *r* is the vector of the distant point as shown in Figure A-2(b). So, magnetic field at that point *P* is determined by:

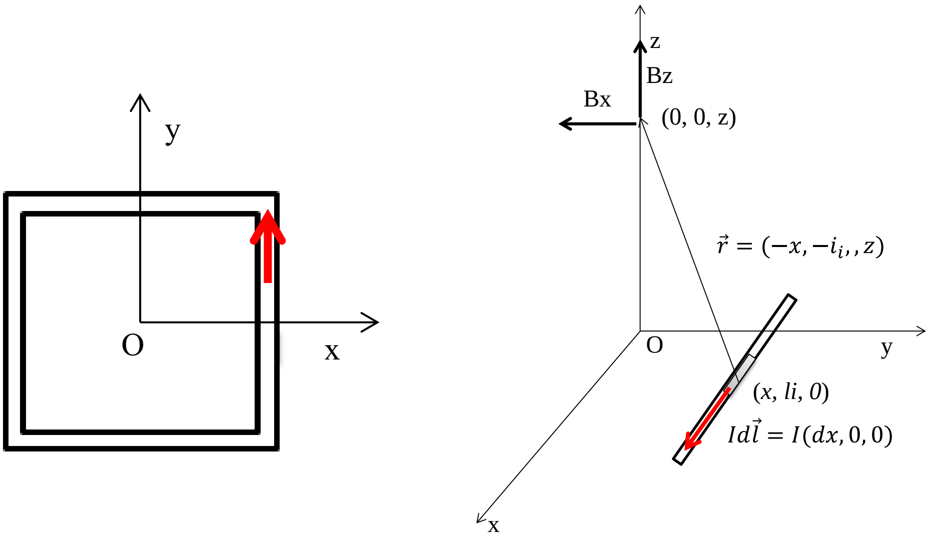

(a) (b)

Figure A-2: (a) Current *I* flow through a single square-shape loop, and (b) the current element *dI* flow through a segment of conducting line.

The current element (*dI*) flowed through a segment of *dl* is displayed as follows:

$$d\bar{I} = Id\vec{l} = Idx\hat{i}(or\ \ Idy\hat{j}) \tag{A-2}$$

The quantities of $\vec{r}$ and $|\vec{r}|$ is then calculated as

$$\bar{r} = (-l_i, -y, z) \tag{A-3}$$

$$|\bar{r}| = \sqrt{l_i^2 + z^2 + y^2} \tag{A-4}$$

Therefore, the outer product of $d\vec{l} \times \vec{r}$ is calculated as

$$d\vec{l} \times \vec{r} = \begin{vmatrix} \hat{i} & \hat{j} & \hat{k} \\ dx & 0 & 0 \\ -x & -l_i & z \end{vmatrix} = -\left(z\hat{i} + l_i\hat{k}\right)dx \tag{A-5}$$

Substituting Eqs.(A-4) and (A-5) into Eq.(A-1), that can be obtained as

$$B_Z = \frac{\mu_o I}{4\pi}\int_{-l_i}^{l_i} \frac{l_i dx_i}{\left(l_i^2 + z^2 + x_i^2\right)^{3/2}} \tag{A-6}$$

Here, x_i represents as x or y. Eq.(A-6) is also expressed as follows:

$$B_Z = \frac{\mu_o I}{4\pi}\int_{-l_i}^{l_i} \frac{l_i dx_i}{\left(l_i^2 + z^2\right)^{3/2}\left(1 + \frac{x_i^2}{l_i^2 + z^2}\right)^{3/2}} \tag{A-7}$$

Suppose $\tan\mu = \frac{x_i}{\sqrt{l_i^2 + z^2}}$ and differentiating both sides of the equation, we can get

$$\sec^2\mu d\mu = \frac{dx_i}{\sqrt{l_i^2 + z^2}} \tag{A-8}$$

Substituting Eq.(A-8) into Eq.(A-7), and then Eq.(A-7) can be obtained as

$$B_Z = \frac{\mu_o I}{4\pi}\int_{-\mu_i}^{\mu_i} \frac{l_i \sqrt{l_i^2 + z^2}\ \sec^2\mu\, d\mu}{\left(l_i^2 + z^2\right)^{3/2}\left(1 + \tan^2\mu\right)^{3/2}} \tag{A-9}$$

Eq.(A-9) can be simplified as

$$B_Z = \frac{\mu_o I}{4\pi}\int_{-\mu_i}^{\mu_i} \frac{l_i\ \sec^2\mu\, d\mu}{\left(l_i^2 + z^2\right)\left(\sec^2\mu\right)^{3/2}} \tag{A-10}$$

Eq.(A-10) can integrated as bellows:

$$B_Z = \frac{\mu_o I}{4\pi}\int_{-\mu_i}^{\mu_i} \frac{l_i\ \cos\mu\, d\mu}{\left(l_i^2 + z^2\right)} = \frac{\mu_o I}{4\pi}\frac{l_i \cdot \sin\mu}{\left(l_i^2 + z2\right)}\Bigg|_{-\mu_i}^{\mu_i} \tag{A-11}$$

Because of $\tan\mu = \frac{x_i}{\sqrt{l_i^2 + z^2}}$, therefore, $\sin\mu = \frac{x_i}{\sqrt{l_i^2 + z^2 + x_i^2}}$. Substituting sin μ into (A-11), Eq.(A-11) can be obtained as

$$B_Z = \frac{\mu_o I}{4\pi}\frac{2l_i^2}{\left(l_i^2 + z^2\right)\left(2l_i^2 + z^2\right)^{1/2}} \tag{A-12}$$

The formula to calculate the magnetic field at any particular point on the of the eight square-shape loops of side length l_i and current carrying I, at a point P far from it at a distance z-axis is given by:

$$B_Z = \frac{4\mu_o I}{4\pi} \sum_{i=1}^{N=8} \frac{2l_i^2}{\left(l_i^2 + z^2\right)\left(2l_i^2 + z^2\right)^{1/2}} \qquad \text{(A-13)}$$
