# Supplementary material for: Magnetic Beads inside Droplets for Agitation and Splitting Manipulation by Utilizing a Magnetically Actuated Platform
Source: Micromachines (Basel). 2023 Jun 30;14(7):1349. doi: 10.3390/mi14071349 (PMC10384566; doi:10.3390/mi14071349)
Supplement: Supplementary file 1 [file micromachines-14-01349-s001.zip › supplementary document S3.pdf]

The 1-D (i.e., z-directional), steady-state heat conduction equation can be expressed by:

$$\frac{\partial}{\partial z}\left(k\frac{\partial T}{\partial z}\right)+\dot{g}=0 \tag{B-1}$$

The power generation density ($\dot{g}$) of coil is obtained as

$$\dot{g}=\frac{\dot{Q}}{V}=\frac{I^2R}{tA_S}=\frac{L_C\times I^2}{t\sigma A_S A_C} \tag{B-2}$$

Here, $\boldsymbol{\sigma}$ and A_C are electric conductivity, and cross-sectional area of a winding copper, respectively. A_S and t are surface area and thinness of a magnetic-actuated chip for a single coil, respectively.

By integrating Eq.(B-1), it can be performed as

$$T(z)=\frac{\dot{g}}{2k}z^2+C_1z+C_2 \tag{B-3}$$

The boundary condition for the center of the chip can be gotten $\left.\frac{dT}{dz}\right|_{z=0}=0$, thus C_1=0. In additional to, the boundary condition for top and bottom surfaces dissipated by heat convection is expressed by

$$\frac{-1}{2}k\nabla T\Big|_{\frac{t}{2}}=h(T_S|_{\frac{t}{2}}-T_\infty) \tag{B-4}$$

Where, k and h are thermal conductivity and heat convention coefficient, respectively.

$$h\left[\left(\frac{\dot{g}}{4k}\left(\frac{t}{2}\right)^2+C_2\right)-T_\infty\right]=k\frac{\dot{g}}{2k}\left(\frac{t}{2}\right) \tag{B-5}$$

C_2 can be obtained as $C_2=T_\infty+\frac{\dot{g}t}{4h}-\frac{\dot{g}t^2}{8k}$. Therefore, Eq.(B-3)

$$T(z)=\frac{\dot{g}}{2k}\left(z^2-\left(\frac{t}{2}\right)^2\right)+\frac{\dot{g}t}{4h}+T_\infty \tag{B-6}$$

The surface temperature of the coil chip can be obtained while z=t/2.

$$T_S=\frac{L_C}{4\sigma hA_SA_C}I^2+T_\infty \tag{B-7}$$
